# Fluctuations in the skeletal muscle power-velocity relationship and interferon-γ after a muscle-damaging event in humans

**DOI:** 10.1186/2046-7648-1-6

**Published:** 2012-10-01

**Authors:** Tyler Barker, Vanessa T Henriksen, Thomas B Martins, Carl R Kjeldsberg, Harry R Hill

**Affiliations:** 1 The Orthopedic Specialty Hospital, 5848 S Fashion Blvd, Murray, UT 84107, USA; 2 ARUP Laboratories, Institute for Clinical and Experimental Pathology, Salt Lake City, UT, 84108, USA; 3 Department of Pathology, University of Utah, Salt Lake City, UT, 84132, USA

**Keywords:** Muscle damage, Interferon-γ, Stretch-shortening contractions

## Abstract

**Background:**

Skeletal muscle power is velocity-dependent under constant load conditions. Interferon (IFN)-γ is an inflammatory cytokine that regulates skeletal muscle recovery following insult in experimental animals. It is unknown if the power-velocity relationship and IFN-γ are modulated after a muscle-damaging event in humans. Therefore, the purpose of this study was to identify the power-velocity relationship and circulating IFN-γ concentration responses to a muscle-damaging event in humans.

**Methods:**

Nine healthy males participated in this study. Each subject had one leg randomly assigned as the control leg. The other leg served as the treatment leg and performed an intense-stretch-shortening cycling (SSC) exercise protocol to induce muscle damage. To measure muscle damage and the power-velocity relationship, unilateral peak isometric force and power output (forces and velocities) measurements were performed prior to, immediately after, and during the days following the SSC protocol. The circulating IFN-γ concentrations were measured in serum samples obtained prior to, immediately after, and during the days following the SSC protocol. Statistical significance of single-leg isometric force and power output data were assessed using a two-way (time and leg treatment) analysis of variance (ANOVA) with repeated measures, followed by a Tukey’s honestly significant difference (HSD) to test multiple pairwise comparisons. The statistical significance of the IFN-γ data were assessed using a one-way (time) ANOVA with repeated measures, followed by a Tukey’s HSD to test multiple pairwise comparisons.

**Results:**

In the treatment leg, significant (*P* < 0.05) peak isometric force deficits occurred immediately and persisted several days after the SSC protocol, thereby identifying muscle damage-induced weakness. During muscle weakness in the treatment leg, peak power was significantly (*P* < 0.05) depressed and the velocities at peak power were significantly (*P* < 0.05) slower. Interestingly, circulating IFN-γ concentrations decreased at 2 and 3 days after compared to those immediately following the SSC protocol.

**Conclusion:**

We conclude that the velocity to achieve a compromised peak power is reduced, and speculatively, the circulating IFN-γ excursion could be influential on the recovery of skeletal muscle after a muscle-damaging event in humans.

## Background

Skeletal muscle power is an important determinant of human locomotion. Skeletal muscle power production is velocity-dependent, and therefore, should be interpreted in relation to the velocity of the movement required to produce peak power [1-3]. Although the power-velocity relationship is a fundamental principle in muscle physiology, surprisingly, human studies examining this relationship during multi-joint movements that resemble locomotive activities are limited, especially during skeletal muscle weakness and soreness induced by muscle damage. Muscular weakness induced by a damaging event (i.e., muscle damage-induced weakness) is a unique measure of rehabilitation because it spans the degenerative and regenerative biological processes that govern recovery [4]. Millions of people worldwide every year are affected by muscle weakness induced by a damaging event, which can be mediated by a variety of conditions, including intense or exhaustive stretch-shortening cycling (SSC) contractions. SSC contractions are characterized by repetitive skeletal muscle lengthening (eccentric) and shortening (concentric) contraction cycles, with the former of the contraction modalities being the dominate mediator of muscle damage. SSC contractions also serve as an excellent model for studying neuromuscular performance [5,6]; in leg muscles, examples of SSC contractions include walking, running, sprinting, and jumping. Compared to ultra-structure disturbances in skeletal muscle and circulating protein alterations, immediate (minutes-to-hours) peak isometric deficits that persist for days, such as those after exhaustive SSC contractions, is a reliable indicator of muscle damage [4].

Inflammatory cytokines are small proteins secreted from nearly every nucleated cell in the body, and importantly, pro- and anti-inflammatory cytokines are essential for skeletal muscle regeneration following a damaging event [7]. Interferon (IFN)-γ is a cytokine that possesses pro-and anti-inflammatory properties [8], and provocatively, its anti-fibrotic and proliferative properties promote muscle healing and the recovery in skeletal muscle function after insult in experimental animals [9,10]. Despite animal studies showing a potential role of IFN-γ on the influence of skeletal muscle recovery, there is little evidence identifying the circulating IFN-γ response to muscle damage in humans [11,12].

Therefore, based on the aforementioned gaps in our knowledge, the primary aim of this study was to identify the power-velocity relationship during persistent peak isometric force deficits induced by intense SSC exercise. We hypothesized that there would be a down- and left-ward shift in the power-velocity relationship during muscular weakness induced by a muscle-damaging event. The secondary aim of this study was to measure circulating IFN-γ alterations after a muscle-damaging event. We hypothesized that IFN-γ would decrease after a muscle-damaging event in humans.

## Results

### Isometric force

**Figure 1:**
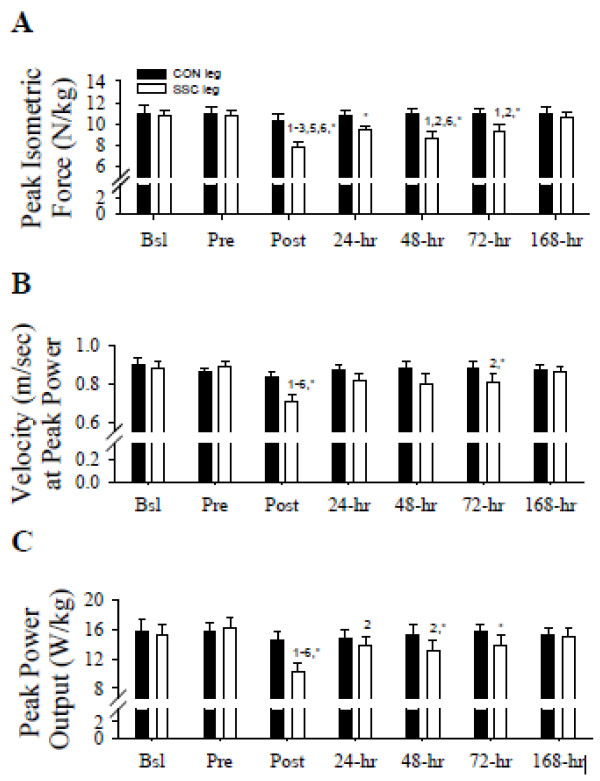
**Peak isometric force, velocity at peak power, and peak power output data.** (**A**) Single-leg peak isometric force (Newton per kilogram), (**B**) velocity at peak power (meters per second), and (**C**) peak power output (watts per kilogram) data prior to and following the SSC protocol 1, *P* < 0.05 vs. Bsl; 2, *P* < 0.05 vs. Pre; 3, *P* < 0.05 vs. 24 h; 4, *P* < 0.05 vs. 48 h; 5, *P* < 0.05 vs. 72 h; 6, *P* < 0.05 vs. 168 h. *P < 0.05 vs. corresponding CON. Legend provided in (**A**). Data presented as mean ± standard error of mean (SEM).

There was a significant (*F* = 13.368 (1,6) *P* < 0.05) treatment per time interaction in peak isometric forces (Newton per kilogram; Figure 1A). Peak isometric forces were not significantly changed in the control (CON) leg. In contrast, peak isometric forces in the SSC leg were significantly decreased (*P* < 0.05) (i.e., Post; approximately 28%) immediately and shortly after the SSC protocol (48 h (approximately 19%) and 72 h (approximately 13%) compared to those prior to the SSC protocol (i.e., Bsl and Pre). Immediately following the SSC protocol (i.e., Post), peak isometric forces were significantly (*P* < 0.05) decreased compared to those at 24, 72, and 168 h. At 48 h peak isometric forces were significantly (*P* < 0.05) decreased compared to those at 168 h in the SSC leg. Furthermore, peak isometric forces in the SSC leg were significantly (*P* < 0.05) decreased at Post (approximately 24%), 24 h (approximately 12%), 48 h (approximately 21%), and 72 h (approximately14%) compared to those in the CON leg. Thus, peak isometric force deficits persisted days after the SSC protocol, thereby indicating the likelihood of muscle weakness induced by muscle damage.

### Velocity at peak power

A significant (*F* = 3.81 (1,6) *P* < 0.05) treatment per time interaction was observed in the velocities (meters per second) at peak power (Figure 1B). Velocities at peak power were not significantly changed in the CON leg. Velocities at peak power were significantly (*P* < 0.05) slower in the SSC leg at Post compared to all other time points. At 72 h, velocities at peak power were significantly (*P* < 0.05) slower compared to those at Pre in the SSC leg. Velocities at peak power were also significantly (*P* < 0.05) different between the SSC and CON legs at Post and 72 h. These data indicate an immediate and a secondary slowing in velocities at peak power after an intense SSC contraction protocol.

### Peak power output

There was a significant (*F* = 8.08 (1,6) *P* < 0.05) treatment per time interaction in peak power outputs (watts per kilogram; Figure 1C). Peak power outputs were not significantly changed in the CON leg. Conversely, peak power outputs were significantly (*P* < 0.05) decreased after (Post, 24, and 48 h) compared to those immediately before (Pre) the SSC protocol in the SSC leg. Immediately after the SSC protocol (Post), peak power outputs were significantly (*P* < 0.05) decreased compared to those at Bsl (approximately 33%), 24, 48, 72, and 168 h in the SSC leg. The SSC leg peak power outputs were significantly (*P* < 0.05) decreased compared to those in the CON leg at Post (approximately 29%), 48 h (approximately 14%), and 72 h (approximately11%).

### Velocity-force and power-velocity relationship

**Figure 2:**
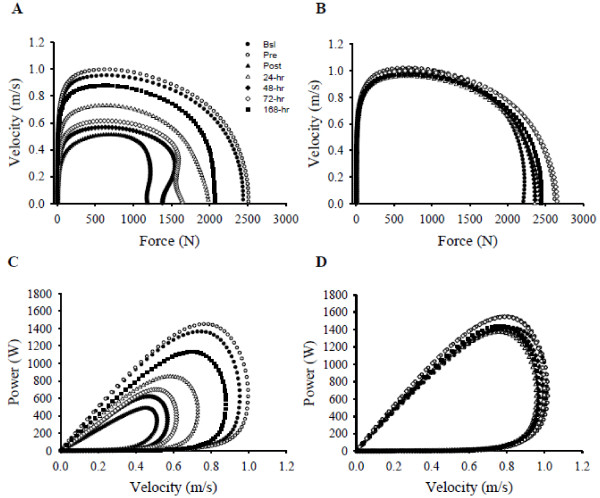
**Example of the concentric velocity-force and power-velocity relationship data.** The concentric force-velocity relationship jump data (collected at 200 Hz with a low-pass filter at 10 Hz) that produced peak power at each time point for the SSC (**A**) and CON (**B**) leg are provided from one representative subject. For a given force (Newton; for example, at 500 N), velocity (meters per second) varied in the SSC (**A**) but not the CON (**B**) leg. Regarding the power-velocity relationship, power (W) varied as a function of velocity in the SSC (**C**) but not the CON (**D**) leg. Velocities at peak powers were decreased after the SSC protocol in the SSC (**C**), but not the CON leg (**D**). Figure legend provided in (**A**).

Examples of the concentric velocity-force and power-velocity relationships for one subject are provided in Figure 2. As illustrated for this subject, but consistent for all subjects (data not shown), velocities at a given submaximal force (e.g., 500 N) were decreased immediately and the first few days after the SSC protocol in the SSC (Figure 2A), but not the CON leg (Figure 2B). Furthermore, velocities at peak powers were decreased after the SSC protocol in the SSC (Figure 2C) but not the CON leg (Figure 2D).

### Perceived muscular soreness

**Figure 3:**
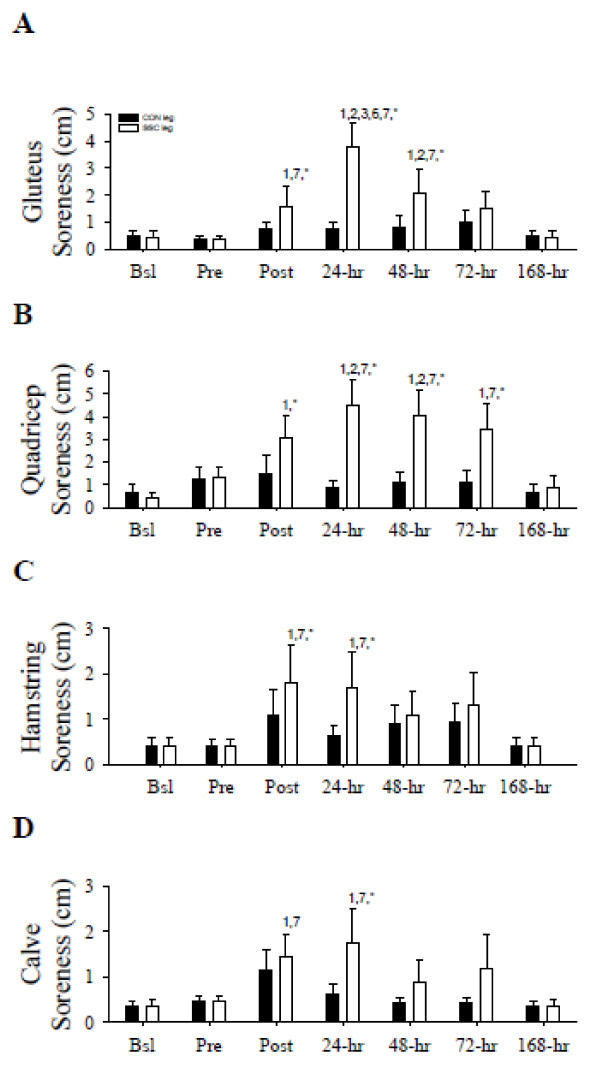
**Perceived level of gluteus, quadricep, hamstring, and calf soreness.** Perceived level of gluteus (**A**), quadricep (**B**), hamstring (**C**), and calf (**D**) soreness (centimeters) prior to and following the SSC protocol. 1, *P* < 0.05 vs. Bsl; 2, *P* < 0.05 vs. Pre; 3, *P* < 0.05 vs. Post; 4, *P* < 0.05 vs. 24 h; 5, *P* < 0.05 vs. 48 h; 6, *P* < 0.05 vs. 72 h; 7, *P* < 0.05 vs. 168 h; *P < 0.05 vs. corresponding CON. Legend provided in (**A**). Data presented as mean ± SEM.

There were significant treatment per time interactions in the gluteus (*F* = 10.54 (1,6) *P* < 0.05; Figure 3A), quadricep (*F* = 11.0 (1,6) *P* < 0.05; Figure 3B), and hamstring (*F* = 2.88 (1,6) *P* < 0.05; Figure 3C) soreness, and significant main effects of time (*F* = 3.55 (1,6) *P* < 0.05) and treatment (*F* = 5.69 (1,6) *P* < 0.05) in calve soreness (Figure 3D). No significant differences in the perceived muscular soreness were observed in the CON leg for any muscle group. However, the perceived level of soreness of the gluteus in the SSC leg was significantly (*P* < 0.05) increased at Post (vs. Bsl and 168 h), 24 h (vs. Bsl, Pre, Post, 72 h and 168 h), and 48 h (vs. Bsl, Pre and 168 h).

Perceived gluteus soreness in the SSC leg was significantly (*P* < 0.05) increased compared to those in the CON leg at Post, 24 and 48 h. Perceived quadricep soreness in the SSC leg was significantly (*P* < 0.05) increased at Post (vs. Bsl), 24 h (vs. Bsl, Pre and 168 h), 48 h (vs. Bsl, Pre and 168 h), and 72 h (vs. Bsl). Soreness in the SSC leg was also significantly (*P* < 0.05) greater than those in the CON leg at Post, 24, 48, and 72 h.

The hamstring soreness was significantly (*P* < 0.05) increased at Post and 24 h (both vs. Bsl and 168 h) in the SSC leg compared to the corresponding values in the CON leg. Perceived calve soreness significantly (*P* < 0.05) increased at Post and 24 h (both vs. Bsl and 168 h) in the SSC leg and in the SSC leg compared to CON leg at 24 h. Taken collectively, the data indicate immediate muscular soreness that persisted for several days after intense SSC contractions.

### Circulating cytokine data

We did not observe a significant change in tumor necrosis factor (TNF)-α (Table 1). However, IFN-γ was significantly (*F* = 3.58 (7) *P* < 0.05) decreased at 48 and 72 h compared to that at Post (Table 1).

**Table 1:** Cytokine concentrations (picograms per milliliter)

	**TNF-α**	**IFN-γ**
Bsl	5.35 ± 2.94	21.5 ± 9.8
Pre	6.84 ± 3.05	21.9 ± 9.8
Post	5.48 ± 2.94	25.4 ± 11.5*
1 h	5.73 ± 2.64	19.6 ± 8.8
24 h	5.77 ± 2.77	19.5 ± 8.6
48 h	4.97 ± 2.19	17.3 ± 7.8
72 h	3.33 ± 1.43	18.3 ± 8.2
168 h	3.89 ± 1.61	22.5 ± 10.3

*Significantly (*P* < 0.05) different from 48 and 72 h; data presented as mean ± SEM.

## Discussion

Previous investigations report power output disturbances during muscle damage-induced weakness [13-20]. In the present report, we extend those findings by identifying concomitant peak power and velocity at peak power decreases during persistent muscular weakness and soreness after intense SSC contractions. These unique and impactful findings identify that the human body sacrifices the speed of a movement to accomplish a diminished power output after a muscle-damaging event. Additionally, we found a small but significant IFN-γ decrease after the SSC protocol, plausibly suggesting that the circulating cytokine excursion could be influential on the recovery of skeletal muscle after a muscle-damaging event in humans.

A methodological novelty of the present investigation was that power outputs were measured under constant load conditions. This enabled us to assess velocity and to extend the velocity-dependent data obtained from isolated muscle research to human movement. Using this methodology, we provide original data identifying velocity at peak power slowing immediately and again 3 days after the SSC protocol (Figure 1B) and during a multi-joint movement. Several investigations previously report various velocity results after intense SSC exercise [14,16,18,21-24]. For example, the take-off velocity decreases during rebound drop jumps from various heights after intense exercise [14,24]. Although these velocities are a close approximation of the velocity at peak power, velocities were not temporally aligned to peak power. Recently, Power et al. [25] provided compelling data illustrating a velocity-dependent power loss following eccentric contractions of the ankle dorsiflexors in humans. However, outcome measures were completed 30 min after the eccentric contractions. Therefore, conclusions regarding velocities at peak power during persistent muscular weakness after a muscle-damaging event have not been explicitly delineated in humans, until now. For the first time, we demonstrate velocity at peak power decreases after a muscle-damaging event in humans. This velocity at peak power decrease occurred concomitantly with a decrease in peak power, and thus, establishing a down- and left-ward shift in the power-velocity relationship following a muscle-damaging event.

The down- and left-ward shift in the power-velocity relationship is a unique finding of this study. Although it is beyond the scope of the present investigation, several fatigue- and damage-related mechanisms could be responsible for the velocity at peak power slowing. First, fatigue-related byproducts (i.e., inorganic phosphate, adenosine diphosphate, and acidosis) increase following a damaging event [26-29] and impair contractile shortening velocity in skeletal muscle fibers obtained from experimental animals [30-32]. Second, low-frequency torque depressions reflect a disruption in excitation-contraction coupling [33,34] and occur with shortening velocity slowing in the human tibialis anterior [25]. Third, fast muscle fibers are regarded as the predominant fiber type susceptible to muscle damage [35,36]. In theory, damage to fast fibers should slow shortening velocity in skeletal muscles composed of heterogeneous fibers. Finally, inflammatory-derived mediators could be modulating velocity after a muscle-damaging event. For example, the pro-inflammatory cytokine, TNF-α, has been found to impede skeletal muscle shortening velocity in experimental animals [37]. However, in the present study, we did not observe a significant TNF-α increase in the circulation, and local concentrations were not measured. Given the preponderance of evidence identifying a localized pro-inflammatory response following a muscle-damaging event [12,33,38,39] and taking into consideration the muscle soreness reported in the SSC leg by our subjects (Figure 3A-D), it is plausible that a localized pro-inflammatory response, among other mediators, is contributing to the velocity impairments during muscular weakness after a damaging event.

An intriguing finding in the present investigation was the subtle fluctuation in IFN-γ days after a muscle-damaging event in humans (Table 1). Few studies have investigated the influence of a muscle-damaging event or exhaustive exercise on IFN-γ. In athletes, serum IFN-γ concentrations remained constant, but in whole blood culture, lipopolysaccharide-induced production of IFN-γ was abolished after exhaustive exercise (sprint triathlon under competitive conditions) [11]. In physically active subjects, serum IFN-γ concentrations were not significantly different following uphill or downhill running [12]. These previous studies suggest no change or decrease in IFN-γ following exhaustive or eccentrically biased exercise [11,12]. Herein, serum IFN-γ concentrations were decreased at 48 and 72 h compared to those immediately after intense SSC exercise (Table 1). Although no significant increase in serum IFN-γ was observed immediately following the SSC exercise, our findings extend previous reports [11,12] by illustrating a time-sensitive decrease in concentrations 2 and 3 days following exercise. This finding warrants additional research for future resolve since this is the first study illustrating a decrease in serum IFN-γ concentrations after an acute muscle-damaging event in humans.

Lymphocytes and natural killer (NK) cells are major sources of IFN-γ [40]. In damaged skeletal muscle, IFN-γ expression is time-sensitive and correlates with the accumulation of T-cells, macrophages, and NK cells [9]. We speculate that the later circulating IFN-γ decrease is the result of immune cells migrating from the circulation to the site of localized trauma in the skeletal muscle after a damaging event.

In skeletal muscle IFN-γ could contribute to the early regenerative process. For example, neutralizing antibodies against the IFN-γ receptor lowered C_2_C_12_ muscle cell proliferation after injury, suggesting that the presence of IFN-γ contributes to proliferation [9]. However, contrasting results exist *in vitro*, which specifically indicate that IFN-γ inhibits C_2_C_12_ and myoblast proliferation and differentiation [41]. These *in vitro* discrepancies could, potentially, be explained by disparate IFN-γ concentrations [42], as low concentrations promote [43] and high concentrations inhibit [44] satellite cell proliferation. Based on the conflicting reports in the literature, it is unclear if the present fluctuations in serum IFN-γ concentrations were beneficial on the skeletal muscle following a damaging event.

There are limitations of this study that require attention. First, subjects performed the SSC contractions as fast as possible and jumped as high as possible under constant load conditions. Under constant load conditions, external resistance is variable through a range of motion, which could alter the precise intensity of the muscle contraction within and between our subjects. Future studies utilizing a dynamometer are encouraged. Secondly, muscle biopsies were not obtained to assess localized microtrauma. However, muscle damage is reliably identified by persistent (days) peak isometric force deficits following intense SSC contractions [13,14,21,24,45-47]. Therefore, based on our peak isometric deficits that persisted for several days (Figure 1A), we believe that our SSC exercise protocol induced muscle damage as opposed to fatigue where peak isometric forces would have recovered rapidly [4]. However, it is likely that our high-intensity exercise protocol, which involved minimal inter-set rest, mediated both muscle fatigue and damage. We speculate that fatigue could have contributed to muscular weakness immediately following the intense exercise protocol, but again, based on the persistent force deficits, damage-related events could be the dominating factors contributing to muscular weakness days after the intense exercise protocol. Next, cytokine data was variable (Table 1). In order to account for this variability statistically, we performed a rank transformation to achieve normality and equal variance. Finally, cytokine data was limited to circulating measures. Future studies are encouraged to examine circulating and local inflammatory cytokine challenges, and to identify if these challenges influence the recovery in skeletal muscle function after a muscle-damaging event.

## Conclusions

Revealing the influence of a muscle-damaging event on the velocity at peak power has significant implications on locomotion, physical performance, and physical rehabilitation in diverse pathophysiological and physiological conditions in humans. In the present investigation, we provide unique results illustrating concomitant decreases in peak power and the velocity at peak power after a muscle-damaging event in humans. This down- and left-ward shift in the power-velocity relationship occurred during a circulating decrease in IFN-γ, an inflammatory cytokine that regulates skeletal muscle recovery. These findings are of clinical and practical relevance because they identify that muscle damage slows the velocity at which a compromised peak power occurs, and importantly, that the recovery in skeletal muscle after a damaging event could be influenced by IFN-γ. Thus, in humans, we conclude that muscle damage mediates a velocity-dependent loss in peak power and that the recovery in skeletal muscle function after a muscle-damaging event could be related to a cytokine that possesses anti-fibrotic and proliferative properties.

## Methods

### Participants

The Institutional Review Board at Intermountain Healthcare (Salt Lake City, UT, USA) approved this study. Subjects were informed of and provided written and verbal consent to the experimental protocol and procedures. Based on our pilot studies and the primary aim of this study, in order to detect an 18% difference between peak power output means within the SSC leg from Pre to 48 h with a statistical power of 90% at an α = 0.05 at least six subjects were required. However, anticipating that some subjects may not complete the protocol, we therefore recruited and enrolled nine healthy, recreationally active (i.e., 30 min of continuous physically active at least three times per week) males (age, 33 ± 2 years; height, 182 ± 2 cm; 89 ± 4.1 kg; body mass index, 26.9 ± 1.1 kg/m^2^) to participate in this study. Subjects were excluded if they were taking any dietary supplements, anti-inflammatory medication(s), and/or suffered a lower leg injury during the previous year that required the use of crutches. Subjects were also excluded if strength asymmetry (>5% difference in peak isometric force or power output) was present between legs. The symmetry in leg strength was important since all strength testing and the SSC protocol were performed unilaterally and statistical comparisons were made within and between legs. Thus, differences in leg strength upon study enrollment could be a confounding factor.

### SSC protocol

Upon study enrollment, each subject had one leg randomly assigned as the CON leg. The other leg served as the treatment (SSC) leg and performed an intense SSC protocol. The rationale for performing this protocol unilaterally was to assess isometric force and power output outcomes within and between legs across time.

**Figure 4:**
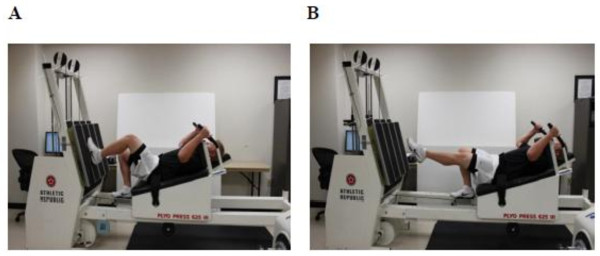
**Example of single-leg strength testing on the horizontal Plyo-press.** (**A**) During single-leg peak isometric testing, the weight stack was overloaded. Overloading the weight stack prevented sled movement, thereby allowing for an isometric contraction. (**B**) Subjects performed repeated jumps during the single-leg jump tests. Illustrated is an example of a concentric Plyo-press jump from push-off (**A**) to peak height (**B**). Weight stack resistance was set at 75% of body mass.

Based on our preliminary experiments to establish a protocol that induces persistent peak isometric force deficits and to measure power output using the same multi-joint modality for inducing muscle damage, each subject performed an SSC protocol that consisted of ten sets of ten repetitive jumps at 75% of body mass with a 20 s rest between each set (Figure 4). Subjects were instructed and verbally encouraged to perform each set with maximal effort and to jump as-high-as possible through a full range of motion (90° of knee flexion-to-full extension) during the SSC protocol. If subjects were no longer able to complete two successive jumps, subjects were then allowed to perform presses through a full range of motion (90° of knee flexion-to-full extension). It should be noted that switching from jumps to presses presumably lowered the eccentric magnitude during loading. If subjects were unable to complete the presses, the exercise protocol was terminated. The mean number of jumps and presses completed during the SSC protocol were 73 ± 10 and 15 ± 5, respectively, while three of the nine subjects were unable to complete the SSC protocol.

### Analytical procedures

#### *Single-leg strength testing*

Single-leg peak isometric force and power output measurements were performed on seven different occasions: 1) baseline (Bsl; 28-d before the SSC protocol); 2) immediately before (Pre) the SSC protocol; and 3) immediately (Post), 4) 24, 5) 48, 6) 72 and 7) 168 h (7-d) after the SSC protocol.

All single-leg strength testing was performed on a horizontal Plyo-press (Athletic Republic, Park City, UT, USA) (Figure 4). Plyo-press output data were measured from signals obtained from a force plate (Advanced Mechanical Technology, Watertown, MA, USA) mounted to the footplate of the Plyo-press and from a displacement transducer (UniMeasure PA-50-NJC, Corvallis, OR, USA) attached to the weight stack. All data were sampled at 200 Hz with a low-pass filter at 10 Hz using DartPower software (Athletic Republic, Park City, UT, USA, version 2.0). Before every testing session, subjects performed a self-selected warm-up, and the mounted force plate was zeroed without any external force (i.e., without anyone touching the mounted force plate) and then load calibrated at 75% of the subjects body mass (i.e., with the subject on the Plyo-press in a fully extended position and the weight stack resistance set at 75% body mass).

For the single-leg isometric force measurements, the Plyo-press sled was adjusted for each subject to align the knee and hip joint flexion angles to 90°, while the knee joint flexion angle subsequently standardized foot position on the force plate across testing days. The Plyo-press sled position was documented and reproduced to achieve the desired knee and hip joint flexion angles at the follow-up visits. Hip and knee extension-isometric contractions were elicited by overloading the weight stack resistance (>2,260 N). Each subject performed three maximal single-leg isometric contractions. The leg selection at the start of each testing session was randomized and subsequently followed by an alternating sequence of leg contractions. Each isometric contraction was 3 s in duration and separated by approximately 1 min of rest. Subjects were verbally instructed and encouraged to exert maximal force against the mounted force platform. Peak isometric force was defined as the highest resultant force applied during the 3 s test and was expressed relative to body mass (Newton per kilogram).

Given the variability in human performance testing and to assess the reproducibility of the effort provided by our subjects, the peak isometric force coefficients of variation at each testing session in each leg were calculated (Additional file 1: Table S1). The coefficient of variations indicated good reliability within each leg at each testing visit. Further, the intra-class reliability coefficient for the peak isometric forces in the CON leg was 0.98, thereby establishing reliability within the CON leg across time. We did not calculate the intra-class reliability coefficient within the SSC leg because peak isometric forces were modulated following the muscle-damaging event, and thus, would influence the reliability calculation.

Single-leg peak power output measures followed the single-leg isometric contractions and were measured on the same horizontal Plyo-press with the same securing procedures described above. Starting from an extended position (full extension = 0°), subjects performed repetitive single-leg jumps (i.e., hip and knee flexion-extension cycles). Subjects were instructed and verbally encouraged to perform the jumps as fast as possible and through a full range of motion (90° of knee flexion-to-full extension). Each test was 20 s (mean number of jumps, 0 jumps/20 s test; range from 9 to 12 jumps/20 s test) in duration with the weight stack resistance set at 75% of body mass (66.2 ± 3.1 kg). The time-aligned product of the resultant force (Newton) acquired from the force platform and weight stack velocity (meters per second) data obtained from the displacement transducer were used to calculate power output. The peak power output was defined as the highest power output produced during the 20 s test for each leg, and the velocity at that peak power was recorded.

To assess the reliability of the jump tests and to determine if subjects were providing consistent effort with each jump at each testing session, the intra-test coefficients of variation for peak powers were calculated for the CON and SSC legs (Additional file 1: Table S1). The intra-test coefficients of variation were good for each leg at each testing visit. The intra-class reliability coefficient for peak power output within the CON leg was 0.96, indicating reliability within the CON leg across time. Similar to peak isometric forces, we did not calculate the intra-class reliability coefficient in the SSC leg because power outputs vary with muscle damage, and are therefore, inconsistent.

#### *Muscle soreness*

Subjects rated their perceived soreness in the gluteus, quadriceps, hamstrings, and calves in each leg. Perceived soreness measures were performed prior to all strength testing sessions and immediately after the SSC protocol. Specifically, subjects rated their perceived level of soreness at Bsl, Pre, Post, 24, 48, 72, and 168 h.

To assess perceived muscular soreness, subjects lowered their bodies into a squat position (90° of hip and knee flexion) for 5 s while resting their back against a wall. This was repeated three times at each visit. In the squat position, subjects rated their perceived level of soreness in the gluteus, quadriceps, hamstrings, and calves on each leg by using a visual analog scale (10 cm in length) of 0 to 10, with 0 being ‘no pain’, 10 being the ‘worst possible pain’ and 5 being ‘tender to touch but not to contractions’ [48]. Subjects made a perpendicular mark on the visual analog scale from which perceived soreness was quantified by measuring to the nearest tenth of a centimeter. The intra-class reliability coefficients for the gluteus, quadriceps, hamstrings, and calves in the CON leg were 0.93, 0.83, 0.90 and 0.93, respectively.

#### *Serum cytokines*

Each subject provided eight fasting blood draws: 1) baseline (Bsl; 28-d before the SSC protocol); 2) immediately before (Pre) the SSC protocol; and 3) immediately (Post), 4) 1 h, 5) 24 h, 6) 48 h, 7) 72 h and 8) 168 h after the SSC protocol. Blood samples were obtained from the antecubital vein into one 6.0 ml serum Becton Dickinson Vacutainer tube (Franklin Lakes, NJ, USA). Serum was separated by centrifugation (VWR International, Radnor, Pennsylvania, Clinical 50 Centrifuge) at 1,068 g for 20 min, within 20 min of sample collection and coagulation. Serum samples were immediately stored at −80°C (Revco Freezer, GC Laboratory Equipment, Asheville, NC, USA) until the day of cytokine concentration analyses. All blood draws were obtained immediately before strength testing, with the exception of the Post draw that was collected after the SSC protocol and after the Post strength testing session.

A multiplex microsphere-bead array was used to measure circulating inflammatory cytokines (picograms per milliliter) as described previously [49,50]. Briefly, serum cytokines were quantitated using a multiplexed sandwich capture assay developed in the ARUP Institute for Clinical and Experimental Pathology (University of Utah, Salt Lake City, UT, USA) using the Luminex Multi-Analyte Profiling system (Luminex, Austin, TX, USA) [49]. Monoclonal capture antibodies for TNF-α and IFN-γ were coupled to microspheres (Luminex). The monoclonal antibody for TNF-α was purchased from Pharmingen/BD Biosciences (San Diego, CA, USA), and IFN-γ was purchased from Biosciences (San Diego, CA, USA). Procedural precision, specificity, and sensitivity of this multiplex-cytokine bead assay have been reported previously [49,50].

#### *Statistical analyses*

To achieve a normal distribution of the data, rank transformations were performed on single-leg strength, muscle soreness, and cytokine data. Transformations were checked for normality with the Kolmogorov-Smirnov test. Statistical significance of the data (single-leg strength and soreness data) was assessed using a two-way (time, leg treatment) analysis of variance (ANOVA) with repeated measures, followed by a Tukey’s honestly significant difference (HSD) to test multiple pairwise comparisons. One-way ANOVA was performed on isometric and power output coefficients of variation within each leg separately (i.e., CON and SSC leg). The one-way ANOVA was used to compute the intra-class reliability coefficients for the single-leg strength testing (peak isometric and power output) and perceived muscular soreness measurements within the CON leg. Statistical significance of the cytokine data (concentrations and concentration changes) were assessed using a one-way (time) ANOVA with repeated measures, followed by a Tukey’s HSD to test multiple pairwise comparisons. Data presented as mean ± SEM. All statistical analyses were performed with SysStat software (SigmaPlot 10.0, SigmaStat 3.5, Chicago, IL, USA). Statistical significance was set at a *P* < 0.05.

## Abbreviations

CON: Control; IFN: Interferon; SSC: Stretch-shortening contractions; TNF: Tumor necrosis factor.

## Competing interests

The authors declare that they have no competing interests.

## Authors’ contributions

TB contributed to study conception and experimental design, data acquisition, analysis and interpretation of data, and the first draft of the manuscript. VTH performed the single-leg strength data analyses. TBM carried out the cytokine analyses. CRK and HRH made significant contributions to the critical revision and intellectual content of this manuscript. All authors read and approved the final version of this manuscript.

## Supplementary Material

Additional file 1 **Table S1.** Intra-test coefficients of variation (%). Click here for file
