# Supplementary material for: Fluctuations in the skeletal muscle power-velocity relationship and interferon-γ after a muscle-damaging event in humans
Source: Extrem Physiol Med. 2012 Oct 1;1:6. doi: 10.1186/2046-7648-1-6 (PMC3707101; doi:10.1186/2046-7648-1-6)
Supplement: Additional file 1 — Table S1. Intra-test coefficients of variation (%). [file 2046-7648-1-6-S1.pdf]

Supplemental Table. Intra-test coefficients of variation (%)

	Peak Isometric Forces		Power Outputs	
	CON leg	SSC leg	CON leg	SSC leg
Bsl	2.36 ± 0.54	3.27 ± 0.39	4.81 ± 0.43	4.12 ± 0.72
Pre	3.90 ± 0.70	4.15 ± 1.02	6.27 ± 0.71	6.03 ± 0.44
Post	NA	NA	5.45 ± 0.52	7.78 ± 1.15
24-hr	3.29 ± 0.48	3.57 ± 0.85	5.49 ± 0.40	6.03 ± 1.01
48-hr	4.68 ± 0.91	4.38 ± 0.69	5.48 ± 0.78	7.81 ± 1.35
72-hr	3.37 ± 0.75	4.42 ± 0.84	4.90 ± 0.66	6.04 ± 0.58
168-hr	3.22 ± 0.33	5.03 ± 1.03	5.93 ± 0.56	4.36 ± 0.59

Data presented as group mean ± SEM.

NA, not available. Coefficients of variation were not computed at Post because only one peak isometric force measurement was performed on each leg at that time point.

No significant differences were observed within the CON or SSC legs.
